# The Removal of H3K27me3 Promoted *SLPI* Transcription and Pubertal Initiation in Pigs

**DOI:** 10.3390/cells15131154

**Published:** 2026-06-25

**Authors:** Yingting He, Ruiqi Wang, Tiantian Wang, Jiahao Shao, Wenmiao Duan, Jinghao Yang, Yuyi Zhong, Xiaolong Yuan, Jiaqi Li

**Affiliations:** 1 State Key Laboratory of Swine and Poultry Breeding Industry, National Engineering Research Center for Breeding Swine Industry, Guangdong Provincial Key Laboratory of Agro-Animal Genomics and Molecular Breeding, College of Animal Science, South China Agricultural University, Guangzhou 510642, China; hyt@scau.edu.cn (Y.H.); scauwrq@stu.scau.edu.cn (R.W.); wtt1018@stu.scau.edu.cn (T.W.); sjh@stu.scau.edu.cn (J.S.); dwmiao@stu.scau.edu.cn (W.D.); stu.jinghao.yang@lglab.ac.cn (J.Y.); zyy@scau.edu.cn (Y.Z.); 2 National Center of Technology Innovation for Pigs, Chongqing 402460, China

**Keywords:** pigs, pubertal initiation, H3K27me3, *SLPI*

## Abstract

**Highlights:**

**What are the main findings?**

- H3K27me3 remodeled the transcriptomic landscape of porcine granulosa cells (GCs), and suppressed GC proliferation by downregulating PCNA and promoted apoptosis by upregulating CASP3, thereby delaying pubertal initiation.
- Loss of H3K27me3 at the *SLPI* promoter during pubertal initiation promoted *SLPI* transcription, enhanced GC proliferation, and suppressed apoptosis, and *Slpi* overexpression in mice accelerated pubertal initiation.

**What are the implications of the main findings?**

- H3K27me3–*SLPI* axis is a developmental stage-specific epigenetic mechanism that governs pubertal initiation in mammals.
- These findings provide novel insights into the epigenetic regulation of female reproduction and identify potential targets for improving reproductive performance.

**Abstract:**

Pubertal initiation critically determines reproductive performance in female pigs. Histone H3 lysine 27 trimethylation (H3K27me3) has been implicated in ovarian development. However, its genome-wide regulatory landscape during the pubertal transition remains unexplored. Here, we obtained transcriptomes of GCs treated with the pharmacological H3K27me3 agonist GSK-J4 or H3K27me3 inhibitor EPZ005687. We found that H3K27me3 substantially remodels the transcriptomic landscape of porcine GCs, with differentially expressed genes significantly enriched in pathways governing cell proliferation and apoptosis. Mechanistically, H3K27me3 suppressed GC proliferation by downregulating the expression of *PCNA* and promoting apoptosis through upregulation of *CASP3*, thereby delaying pubertal initiation. Furthermore, genome-wide ChIP-seq analysis on porcine ovaries from pre-pubertal and in-pubertal gilts revealed higher H3K27me3 enrichment around transcription start sites in the In-puberty stage than in the Pre-puberty stage. Genes with promoters exhibiting reduced H3K27me3 occupancy during the pubertal transition were enriched in pathways related to sex differentiation and serine-type endopeptidase inhibitor activity. Notably, secretory leukocyte peptidase inhibitor (*SLPI*) was identified by ChIP-qPCR as a direct target repressed by H3K27me3. Functional validation demonstrated that *SLPI* promoted GC proliferation and inhibited GC apoptosis in vitro. Intraperitoneal injection of LV-*Slpi* or sh-*Slpi* into C57BL/6J mice showed that *Slpi* accelerated pubertal initiation of mice in vivo. Collectively, our findings confirmed that developmental stage-specific loss of H3K27me3 at the *SLPI* promoter derepressed *SLPI* transcription, which in turn promoted porcine GC proliferation, suppressed apoptosis, and facilitated pubertal initiation in mice. These results provided valuable insights into the epigenetic regulation of pubertal initiation in mammals.

## 1. Introduction

Pubertal initiation is a critical developmental stage marked by the onset of first estrus and ovulation, establishing reproductive capacity [1,2]. In gilts, precocious puberty correlates with enhanced reproductive longevity and increased lifetime parity [3], whereas delayed puberty results in reduced live piglet production across the first three litters [4]. Stancic et al. [5,6] reported that approximately 14% of gilts fail to develop mature follicles by 240 days of age in a commercial farm. Excessive granulosa cell (GC) apoptosis and follicular atresia are major causes of delayed or failed puberty, leading to culling and substantial economic losses [7]. GCs play a crucial role in follicular development by secreting estradiol (E2) [8], which suppresses GC apoptosis [9]. Indeed, disruption of estrogen signaling in mice promotes GC apoptosis [10,11], inhibits folliculogenesis [12], and leads to ovulatory dysfunction and delayed puberty initiation [13]. Despite its critical importance, pubertal initiation in gilts has received comparatively less attention than other economically significant reproductive traits, including litter size and daily weight gain.

Epigenetic modifications, particularly histone post-translational modifications, dynamically regulate gene transcription by modulating chromatin accessibility [14,15]. Histone H3 lysine 27 trimethylation (H3K27me3) is a major post-translational modification to repress gene transcription by promoting a compacted chromatin structure, thereby marking transcriptionally silent genomic regions [16,17]. A well-established example is PRC2-mediated H3K27me3 at the *Kiss1* promoter, which represses *Kiss1* expression and delays pubertal initiation in rats [18]. In mouse ovaries, heat stress-induced elevation of H3K27me3 levels significantly suppresses *Cyp19a1* expression, leading to ovarian dysfunction [19]. In porcine GCs, H3K27me3 promotes *CYP1A1* transcription while suppressing proliferation and facilitating ferroptosis [20]. Elevated levels of H3K27me3 impair oocyte maturation in the polycystic ovary syndrome follicles of humans [21]. Collectively, recent studies have demonstrated that H3K27me3, as a key epigenetic regulatory mechanism, is evolutionarily conserved in mammals. Despite these insights, the mechanisms by which H3K27me3 regulates pubertal initiation in pigs remain largely unexplored.

Our previous work on DNA methylation identified matrix metalloproteinase 2 (MMP2) as a critical regulator of porcine follicular development and pubertal progression. *MMP2* degrades the basement membrane and remodels the extracellular matrix, which essential for GC function [22,23]. Secretory leukocyte peptidase inhibitor (*SLPI*) is known to modulate MMP activity. *SLPI* promotes *MMP2* via the Elk-1 signaling pathway to facilitate gastric cancer cell metastasis [24]. More importantly, compared to normal ovaries, *SLPI* transcription is significantly elevated in GCs of patients with polycystic ovary syndrome [25]. Moreover, *SLPI* inhibits the transcription of hyaluronidase 1, thereby promoting hyaluronic acid synthesis in human ovaries [26]. *SLPI* also promotes the expression of *MMP9* [27], which may influence the apoptosis and migration of ovarian GCs. Despite these insights, the precise mechanisms by which *SLPI* regulates pubertal initiation, follicular development, and GC function remain largely unknown.

To elucidate the mechanisms by which H3K27me3 regulates pubertal initiation, we treated porcine GCs in vitro and mice in vivo with an H3K27me3 agonist or inhibitor to explore the effects of H3K27me3 on follicular development and pubertal timing. Integrated analysis of RNA-seq data from porcine GCs and ChIP-seq data from porcine ovaries identified a novel H3K27me3-*SLPI* axis. We then investigated the molecular mechanisms of H3K27me3-dependent *SLPI* regulation. We also examined how *SLPI* influenced cell proliferation and apoptosis in GCs. The functional impact of *SLPI* on cell proliferation, apoptosis, follicular development, and pubertal initiation was also experimentally validated in porcine GCs in vitro and mice in vivo. We employed a mouse model for in vivo functional assessment, as the mouse is a widely accepted mammalian model for studying reproductive development due to its short reproductive cycle, well-established genetic tools, and defined pubertal initiation endpoints. Our findings revealed that H3K27me3 repressed the transcription of *SLPI* by enriching its promoter, thereby delaying pubertal initiation.

## 2. Materials and Methods

### 2.1. Animals

Six Landrace × Large White gilts were purchased from Baishi Pig Farm (Zhongshan, Guangdong, China) and randomly divided into Pre- and In-puberty groups (*n* = 3 per group). Pre-puberty gilts (162 ± 3 d, 81.38 ± 2.40 kg) were defined by the absence of any pubertal signs (no vulval reddening, swelling, or standing reflex), while In-puberty gilts (212 ± 14 d, 110.00 ± 2.00 kg) were identified by the presence of first pubertal signs. Ovaries with a diameter of ≥5 mm were rapidly removed after euthanasia, immediately frozen in liquid nitrogen, and kept at −80 °C until further processing. Immature follicles used in this study were those ≤5 mm in diameter from pre-puberty gilts, whereas mature follicles are those >9 mm in diameter from In-puberty gilts.

Thirty-five three-week-old female C57BL/6J mice, obtained from the Guangdong Medical Laboratory Animal Center (Guangzhou, China), were randomly allocated into seven experimental groups (*n* = 5 per group): Agonist, Inhibitor, Control, LV-*Slpi*, LV-NC, sh-*Slpi*, and sh-NC. Mice were intraperitoneally injected three times a week for three weeks with H3K27me3 agonist GSK-J4 (6 mM; HY15648B, MCE, Monmouth Junction, NJ, USA), the H3K27me3 inhibitor EPZ005687 (6 mM; HY-15555, MCE, Monmouth Junction, NJ, USA), or 6 mM DMSO (control). GSK-J4 upregulated H3K27me3 levels by inhibiting the H3K27me3 demethylases JMJD3 and UTX [28,29]. EPZ005687 downregulated H3K27me3 levels by inhibiting the methyltransferase EZH2 [30,31]. Lentiviral vectors (1  ×  10^7^ TU; Dongze Biotech, Guangzhou, China) were injected once per week over the same three-week period into the LV-*Slpi*, LV-NC, sh-*Slpi*, and sh-NC groups, following our previous protocol [32]. From each group, three mice were randomly selected for subsequent analyses. The randomization sequence was generated using Microsoft Excel software. The animals were housed in temperature-controlled rooms with ad libitum access to water and food. Anesthesia was induced by intraperitoneal injection of 10% chloral hydrate at a dose of 5 mL/kg body weight.

### 2.2. Cell Culture and Treatments

Porcine ovaries came from a local slaughterhouse and were kept in phosphate-buffered saline (PBS) supplemented with penicillin/streptomycin. To obtain GCs, we punctured follicles (3–5 mm in diameter) by aspirating follicular fluid using a syringe, followed by rinsing with PBS. The harvested GCs were grown in DMEM supplemented with 10% FBS and maintained at 37 °C in 5% CO_2_. For transfection, we used Lipofectamine^TM^ 3000 Reagent (Thermo, Waltham, MA, USA). GCs were treated with GSK-J4 (2 μM) or EPZ005687 (10 μM), and an equal volume of DMSO. The selected concentrations were based on our previous study, which demonstrated that 2 μM agonist GSK-J4 significantly increased the protein level of H3K27me3, and 10 μM inhibitor EPZ005687 significantly decreased the protein level of H3K27me3 in GCs [33]. This experiment was independently repeated two times.

### 2.3. RNA-Seq and Data Analysis

RNA-seq was carried out by Gene Denovo Biotechnology (Guangzhou, China) as previously described [32]. Total RNA was isolated from samples with TRIzol (Invitrogen, Carlsbad, CA, USA). After quality control and library construction, paired-end sequencing was performed on an Illumina NovaSeq 6000 platform. The resulting reads were mapped to the pig reference genome (Sscrofa11.1) using HISAT2 [34]. Transcripts were assembled and quantified with StringTie [35], and DESeq2 was applied for differential expression analysis [36]. Differentially expressed genes (DEGs) were identified with the thresholds of |log2 (fold change)| > 1 and an adjusted *p*  < 0.05. Heatmaps were generated to show hierarchical clustering of DEGs patterns.

### 2.4. ChIP-Seq and Data Analysis

ChIP-seq was conducted by KangChen Biotech (Shanghai, China). Two biological replicates per stage (Pre- and In-puberty) were analyzed, each from a different gilt. Cells were cross-linked with formaldehyde, stopped with glycine, and lysed. Chromatin was sonicated to 200–500 bp fragments and immunoprecipitated overnight using an anti-H3K27me3 antibody (Abcam, Cambridge, UK). Immunoprecipitated DNA was purified and used for library preparation with the TruSeq Nano DNA Sample Prep Kit (Illumina, San Diego, CA, USA). Libraries were size-selected for 200–1500 bp using AMPure XP beads, quantified by an Agilent 2100 Bioanalyzer (Agilent Technologies, Santa Clara, CA, USA), and sequenced on an Illumina HiSeq 4000 platform. After Solexa CHASTITY quality filtering, raw reads per sample ranged from 16.64 M to 24.62 M. Reads were aligned to the pig reference genome (susScr3) using Bowtie2 [37]. Peak calling was performed with MACS 2, with a *p*-value threshold of 1 × 10^−4^ and retaining peaks with fold enrichment ≥ 2. FRiP scores were calculated as uniquely mapped reads overlapping MACS2-called peaks (q < 0.05) divided by total uniquely mapped reads per IP sample. Biological reproducibility was assessed by pairwise Spearman correlation and IDR analysis (IDR < 0.05) following ENCODE guidelines. For visualization and cross-sample comparison, bigWig files were generated using deepTools bamCoverage with Reads Per Genomic Content normalization [38]. Read counts within ±3 kb of transcription start sites were quantified with deepTools. Differential peaks were identified with |log_2_(fold change)| > 1 and adjusted *p* < 0.05.

### 2.5. Plasmid Construction

Based on the Sus scrofa *SLPI* gene sequence in NCBI (NM_213870.1), specific primers were designed to amplify the target fragment. PCR used the PrimerSTAR^®^ high-fidelity enzyme (TaKaRa, Dalian, China). The PCR products were gel-purified, A-tailed, and ligated into the PMD-18T vector. The inserted fragment was subsequently verified by Sanger sequencing, and its sequence was confirmed using DNASTAR software (DNASTAR Inc., Madison, WI, USA). The *SLPI* gene was then cloned into the eukaryotic expression vector pcDNA3.1, which had been digested with the same restriction enzymes.

### 2.6. RNA Extraction and Quantitative Real-Time PCR (qRT-PCR)

Total RNA was isolated with TRIzol (Invitrogen, Carlsbad, CA, USA) and then reverse-transcribed into cDNA by a RevertAid First Strand cDNA Synthesis Kit (Thermo, Waltham, MA, USA). Gene expression was measured by a SYBR Green qRT-PCR Master Kit on the Bio-Rad CFX96 real-time PCR system (Bio Rad, Hercules, CA, USA). Relative mRNA expression was calculated using the 2^−ΔΔct^ method. Each qRT-PCR reaction was independently repeated twice. The primer sequences used are provided in Table 1.

### 2.7. Western Blot Analysis

We followed the Western blot procedure as before [39]. Proteins were separated by SDS-PAGE. We then routinely cut the gel according to the molecular weight of the target protein and transferred the separated proteins onto polyvinylidene difluoride membranes. The membranes were blocked with skimmed milk, then incubated overnight at 4 °C overnight with the following antibodies: H3K27me3 (86992-1-RR, Proteintech, Wuhan, China), PCNA (10205-2-AP, Proteintech, Wuhan, China), Caspase3 (66470-2-Ig, Proteintech, Wuhan, China), Caspase7 (13423-1-AP, Proteintech, Wuhan, China), *SLPI* (17193-1-AP, Proteintech, Wuhan, China), and GAPDH (ab8245, Abcam, Cambridge, UK), and Tubulin (11224-1-AP, Proteintech, Wuhan, China). After adding HRP-conjugated secondary antibodies, we visualized the bands and quantified them with ImageJ software (version 1.8.0_345, National Institutes of Health, Bethesda, MD, USA). The Western blot was independently repeated two times.

### 2.8. EdU Assay

EdU assay was performed using the Cell-Light^TM^ Edu Kit (Guangzhou RiboBio Co., Ltd., Guangdong, China). Cells seeded in 48-well plates were transfected with plasmid for 24 h, then treated with 100 μL of 50 μM EDU for 2 h. After fixation with 4% paraformaldehyde (30 min), permeabilization with 0.5% Triton X-100 (10 min), and staining with Apollo and Hoechst (30 min in the dark), images were captured by a Nikon ECLIPSE Ti2 fluorescence microscope (Nikon, Tokyo, Japan). Three random fields per well were counted. The assay was performed twice independently.

### 2.9. Apoptosis Assay

Apoptosis was evaluated using an Annexin V-fluorescein isothiocyanate (FITC)/propidium iodide (PI) detection kit (BioVision, Milpitas, CA, USA). Cells seeded in 6-well plates were transfected for 24  h, then harvested and resuspended in 1× Annexin V-FITC buffer. Each sample was stained with Annexin V-FITC and PI for 15 min in the dark, then analyzed on a BD FACS Calibur flow cytometer (BD Biosciences, San Jose, CA, USA). In the resulting dot plot, viable cells appear in the lower left quadrant, early apoptotic cells in the lower right, late apoptotic cells in the upper right, and necrotic cells in the upper left. The total apoptosis rate was defined as the sum of early and late apoptotic percentages. The experiment was independently repeated twice.

A TUNEL Apoptosis Assay Kit (Beyotime, Shanghai, China) was used for TUNEL staining. Paraffin-embedded ovarian sections were deparaffinized in xylene (10 min), rehydrated in ethanol (5–10 min), and treated with protease K for 15 min. After being washed with PBS, sections were incubated with TUNEL reaction mixture for 1 h in the dark and imaged by a Nikon ECLIPSE Ti2 fluorescence microscope.

### 2.10. ELISA Detection

The mouse GnRH (CSB-E08152m, Cusabio, Wuhan, China), FSH (CSB-E06871m, Cusabio, Wuhan, China), LH (BES01459K, Bioesn, Shanghai, China), and E2 ELISA Kit (CSB-E05109m, Cusabio, Wuhan, China) were used. Next, 50 μL of standards or samples was added to wells along with 100 μL of HRP conjugate. The mixture was then incubated for 60 min at 37 °C. After washing, 50 μL of substrates A and B were added and incubated for 15 min. The OD value at 450 nm was obtained. Each ELISA was independently repeated two times.

### 2.11. ChIP-qPCR

ChIP-qPCR was performed with the Pierce™ Kit (Thermo, Waltham, MA, USA). Pre- and in-pubertal pig ovaries were cross-linked at room temperature, quenched with glycine, lysed, and sonicated. A fraction of sheared chromatin was kept as input. The rest were incubated overnight at 4 °C with 2 μg of anti-H3K27me3 or control IgG (MilliporeSigma, Burlington, MA, USA). Complexes were captured on protein A/G magnetic beads, washed, and eluted. After reverse cross-linking (proteinase K, 65 °C, 2 h), DNA was purified. qPCR was performed using the SYBR Green Master Mix (Vazyme, Nanjing, China). H3K27me3 enrichment at the *SLPI* promoter was calculated as the percentage of input normalized to a negative control region. Each experiment was performed with three independent biological replicates.

### 2.12. Hematoxylin and Eosin (HE) Staining

The tissue sections were baked in a thermostat and dewaxed in xylene I and xylene II. The sections were immersed in 95% (*v*/*v*) ethanol and 75% (*v*/*v*) ethanol for 5 min each to remove the xylene and then rinsed with distilled water. Next, the sections were immersed in hematoxylin, followed by rinsing with tap water until the color turned blue. The sections were stained with eosin for 3 min, dehydrated with 95% (*v*/*v*) and 100% (*v*/*v*) ethanol for 5 min each, and soaked in xylene I and xylene II. Finally, excess xylene on the glass slide was wiped off, and an appropriate amount of gum was added to seal and cover the slide. Stained sections were imaged by a Nikon ECLIPSE Ti2 microscope. Ovarian follicles are the fundamental functional units of the mammalian ovary, each consisting of an oocyte surrounded by somatic GCs. Preantral follicles (PF) were identified by an oocyte surrounded by multiple GC layers without an antrum. Antral follicles (AF) were defined by a distinct fluid-filled antrum separating mural and cumulus GCs, with the oocyte in the cumulus compartment. Corpora lutea (CL) were characterized by irregular clusters of luteal cells, with occasional irregular cavities and an absence of oocytes. The number of PF, AF, and CL was counted per ovary. Follicles were classified morphologically.

### 2.13. Statistical Analysis

Data are presented as the mean ± s.e.m. from three independent biological replicates. Statistical significance between two groups was determined using an unpaired two-tailed Student’s *t*-test. *p* values were stated in the figures. *, *p* < 0.05. **, *p* < 0.01.

## 3. Results

### 3.1. The Transcriptome of GCs Regulated by H3K27me3

**Figure 1:**
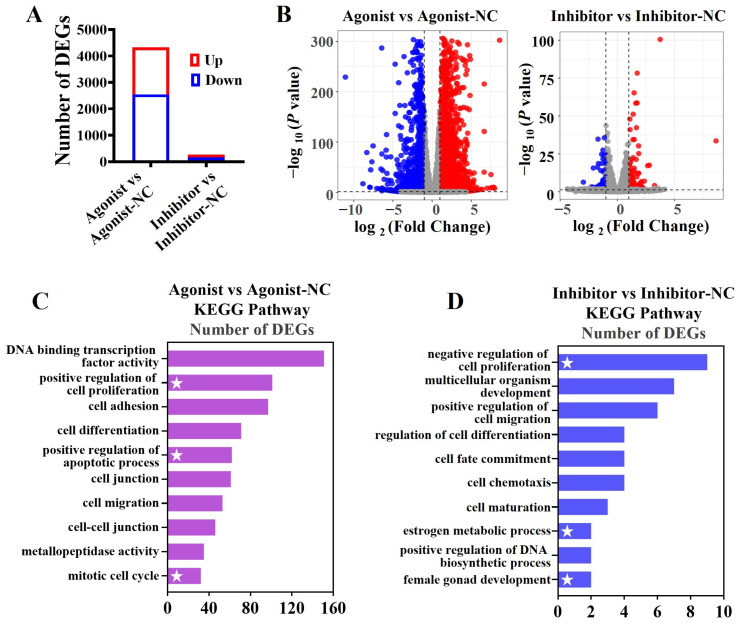
H3K27me3 regulated the transcriptome of GCs in pigs. (**A**) The histogram of DEGs in GCs treated with the H3K27me3 agonist or inhibitor compared to their respective negative controls. (**B**) The volcano plot of DEGs from GCs treated with agonist vs. agonist-NC and inhibitor vs. inhibitor-NC. Red and blue dots denote significantly upregulated and downregulated genes, respectively; gray dots represent genes with no significant differential expression. KEGG pathway analysis of DEGs between Agonist vs. Agonist-NC (**C**) and Inhibitor vs. Inhibitor-NC (**D**) groups. The white star indicates the pathway that we are particularly interested in. Data were from three independent biological replicates.

To investigate the effects of H3K27me3 on GCs, porcine GCs were treated with H3K27me3 agonist (GSK-J4) or inhibitor (EPZ005687). Compared with the Agonist-NC, the agonist-treated group showed 1782 upregulated and 2547 downregulated genes. The inhibitor-treated group exhibited 111 upregulated and 164 downregulated genes compared to its control (Figure 1A). The volcano plot (Figure 1B) depicted the distribution of DEGs. KEGG pathway analysis revealed that the DEGs between the Agonist and Agonist-NC groups were significantly enriched in pathways related to positive regulation of cell proliferation, positive regulation of apoptotic process, and mitotic cell cycle (Figure 1C). DEGs between the Inhibitor and Inhibitor-NC groups were significantly enriched in pathways related to negative regulation of cell proliferation, estrogen metabolic process, and female gonad development (Figure 1D). These results suggest that H3K27me3 modification might alter gene expression and influence cell proliferation and apoptosis in GCs.

### 3.2. H3K27me3 Inhibited Cell Proliferation and Promoted Apoptosis in GCs

**Figure 2:**
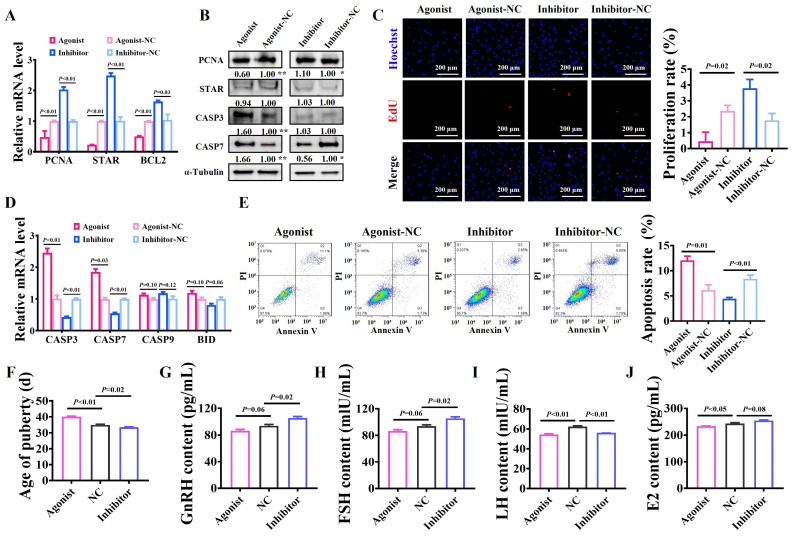
H3K27me3 affected GC proliferation and pubertal initiation. (**A**) The *PCNA*, *STAR*, and *BCL2* mRNA in GCs treated with H3K27me3 agonist or inhibitor. (**B**) The PCNA, STAR, CASP3, and CASP7 proteins in GCs treated with agonist or inhibitor. (**C**) Edu showing the cell proliferation of GCs treated with H3K27me3 inhibitor and agonist. Scale bar = 200 μm. (**D**) The *CASP3*, *CASP7*, *CASP9*, and *BID* mRNA in GCs treated with H3K27me3 agonist or inhibitor. (**E**) Annexin V/PI showing the cell apoptosis of GCs treated with H3K27me3 agonist or inhibitor. (**F**) The ages of pubertal initiation of mice treated with H3K27me3 agonist, inhibitor, or NC (DMSO). The secretion of GnRH (**G**), FSH (**H**), LH (**I**), and E2 (**J**) of mice injected with H3K27me3 agonist, inhibitor, or NC. Data are shown as mean ± SEM from three independent biological replicates. *p* values are stated in the figures. *, *p* < 0.05. **, *p* < 0.01.

To investigate the effects of H3K27me3 on cell proliferation and apoptosis, GCs were also treated with H3K27me3 agonist or inhibitor. Compared to the Agonist-NC group, treatment with the agonist significantly downregulated the mRNA levels of *PCNA*, *STAR*, and *BCL2* (Figure 2A) and the protein levels of PCNA (Figure 2B). Agonist significantly upregulated the mRNA expression of *CASP3* and *CASP7* (Figure 2D) and the protein levels of CASP3 and CASP7 (Figure 2B), which was accompanied by a significant inhibition of proliferation (Figure 2C) and promotion of apoptosis (Figure 2E) in GCs. Conversely, inhibition of H3K27me3 significantly increased *PCNA*, *STAR*, and *BCL2* (Figure 2A) and decreased *CASP3* and *CASP7* (Figure 2D) in mRNA levels. The inhibitor significantly upregulated PCNA and downregulated the CASP7 protein (Figure 2B). In parallel, these molecular shifts led to enhanced proliferation (Figure 2C) and suppressed apoptosis in GCs (Figure 2E). To further investigate the role of H3K27me3 in regulating follicular development in vivo, we intraperitoneally injected the agonist, inhibitor, and NC into C57BL/6J mice. Compared to the controls (35.0 ± 0.5 d), mice injected with the agonist (40.0 ± 0.7 d) showed a significant delay in vaginal opening and pubertal initiation (Figure 2F) and inhibition of the secretion of LH (Figure 2I) and E2 (Figure 2J). Mice injected with the inhibitor (33.7 ± 0.4 d) exhibited a significantly earlier onset of puberty compared with the control group (Figure 2F) and showed promotion of GnRH (Figure 2G), FSH (Figure 2H), and LH (Figure 2I) secretion. Taken together, these findings implied that H3K27me3 markedly inhibited proliferation, promoted apoptosis, and delayed pubertal initiation.

### 3.3. H3K27me3 Participates in Porcine Pubertal Initiation

**Figure 3:**
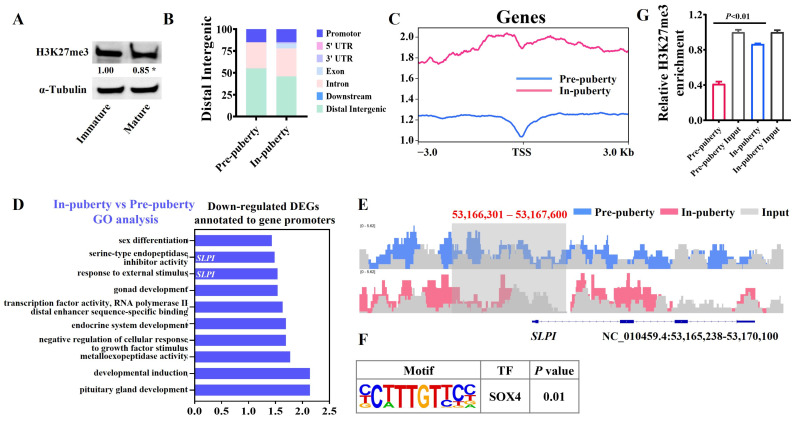
H3K27me3 inhibited *SLPI* transcription. (**A**) The protein levels of H3K27me3 in immature (≤5 mm) and mature follicles (>9 mm) (*n* = 3 per group). (**B**) Distribution of genomic features in Pre-puberty and In-puberty stages (*n* = 2 per group). (**C**) Metagene analysis of normalized H3K27me3 ChIP-seq signal intensity plots for all genes  within ±3 kb of the transcriptional start site. (**D**) GO analysis of downregulated DEGs annotated to gene promoters between In-puberty and Pre-puberty. (**E**) Integrated analysis of ChIP-seq data of *SLPI* promoters in Pre-puberty and In-puberty stages. (**F**) The sequence information and motif analysis of the *SLPI* promoter. (**G**) ChIP-qPCR validation of H3K27me3 enrichment at the SLPI promoter (*n* = 3 per group). *p* values are stated in the figures. *, *p* < 0.05.

We found that the levels of H3K27me3 were significantly higher in immature follicles than in mature follicles (Figure 3A). Considering that H3K27me3 might regulate follicular growth by remodeling chromatin accessibility of key genes, we further performed H3K27me3 ChIP-Seq. We performed peak calling to identify H3K27me3 peaks and characterized their distribution across different stages. The genomic regions modified by H3K27me3 were classified into seven regions: promoter, 5′UTR, 3′UTR, exon, intron, downstream, and intergenic. The H3K27me3 peaks were primarily located in the intron and intergenic regions (Figure 3B). In addition, H3K27me3 enrichment around the transcription start site was higher in the In-puberty group compared to the Pre-puberty group (Figure 3C). Peaks that were identified as differentially enriched and located within gene promoter regions were selected for subsequent analysis. GO analysis of genes associated with differential H3K27me3 promoter peaks between Pre- and In-puberty stages were significantly enriched in sex differentiation, serine-type endopeptidase inhibitor activity, and response to external stimulus (Figure 3D). Moreover, compared to Pre-puberty, the *SLPI* promoter region (chr17: 53,166,301–53,167,600) in the In-puberty group exhibited a significantly lower enrichment of H3K27me3 in the porcine ovary (Figure 3E), and SOX4 might be the potential transcription factor significantly enriched in the *SLPI* promoter (Figure 3F). To investigate the H3K27me3 occupancy at the promoter of *SLPI* during puberty initiation, we performed ChIP-qPCR and found that H3K27me3 enrichment at the *SLPI* promoter was significantly reduced in in-pubertal ovaries compared to pre-pubertal ovaries (Figure 3G). Collectively, these findings indicated that H3K27me3 regulated porcine pubertal initiation by inhibiting *SLPI* transcription.

### 3.4. SLPI Promoted Cell Proliferation and Inhibited Apoptosis in GCs

**Figure 4:**
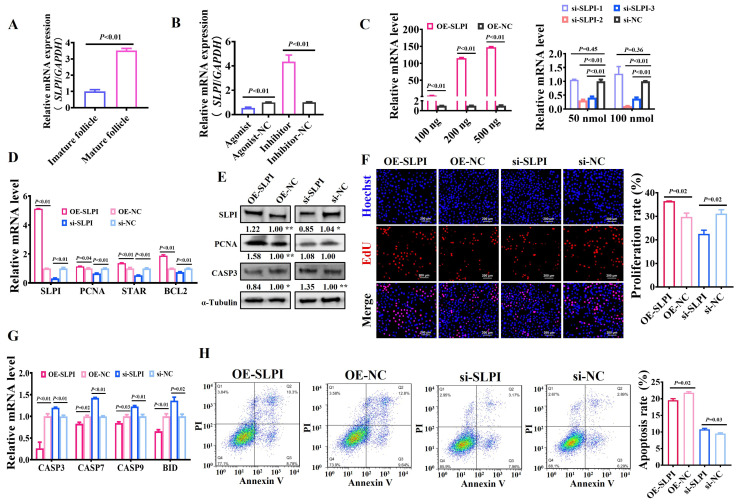
*SLPI* promoted GC proliferation and inhibited apoptosis. (**A**) Comparative analysis of *SLPI* mRNA expression in immature (≤5 mm) and mature ovarian (>9 mm) follicles. (**B**) The mRNA levels of *SLPI* in GCs treated with H3K27me3 agonist or inhibitor. (**C**) The mRNA levels of *SLPI* in GCs transfected with overexpression vectors (OE-*SLPI*) and small interfering RNA (si-*SLPI*). (**D**) The mRNA levels of *SLPI*, *PCNA*, *STAR*, and *BCL2* in GCs treated with OE-*SLPI* and si-*SLPI* for *SLPI*. (**E**) The SLPI, PCNA, and CASP3 proteins in GCs treated with OE-*SLPI* and si-*SLPI*. (**F**) Edu showing the cell proliferation of GCs treated with OE-*SLPI* and si-*SLPI*. Scale bar = 200 μm. (**G**) The mRNA levels of *CASP3*, *CASP7*, *CASP9*, and *BID* in GCs treated with OE-*SLPI* and si-*SLPI*. (**H**) Annexin VI/P showing the cell apoptosis of GCs treated with OE-*SLPI* and si-*SLPI*. Data are shown as mean ± SEM from three independent biological replicates. *p* values are stated in the figures. *, *p* < 0.05. **, *p* < 0.01.

The transcriptional level of *SLPI* was significantly promoted in mature follicles compared to immature follicles (Figure 4A). We observed that H3K27me3 enrichment inversely correlated with *SLPI* transcription, its upregulation in GCs decreased *SLPI* transcription, and its reduction enhanced transcriptional activity (Figure 4B). To elucidate the functional role of *SLPI* in GC biology, overexpression vectors (OE-*SLPI*) and small interfering RNA (si-*SLPI*) were transfected into GC. OE-*SLPI* and si-*SLPI* significantly elevated or reduced the mRNA (Figure 4C) and protein (Figure 4E) levels of *SLPI*, respectively. For subsequent experiments, we used 100 ng of OE-*SLPI* and 50 nmol of si-*SLPI*-1. Compared to the control, *SLPI* overexpression significantly increased the expression of *PCNA*, *STAR*, and *BCL2* in mRNA (Figure 4D) and PCNA in protein (Figure 4E), and it was accompanied by promoted proliferation in GCs (Figure 4F). si-*SLPI* significantly downregulated the expression of *PCNA*, *STAR*, and *BCL2* in mRNA (Figure 4D) and GC proliferation (Figure 4F). Furthermore, OE-*SLPI* significantly inhibited the expression of *CASP3*, *CASP7*, *CASP9* and *BID* mRNA (Figure 4G) and CASP3 protein (Figure 4E) and apoptosis (Figure 4H). In contrast, si-*SLPI* produced the opposite effect. These results demonstrated that *SLPI* promoted proliferation and inhibited apoptosis in porcine ovarian follicles.

### 3.5. Slpi Promoted the Pubertal Initiation in Mice

**Figure 5:**
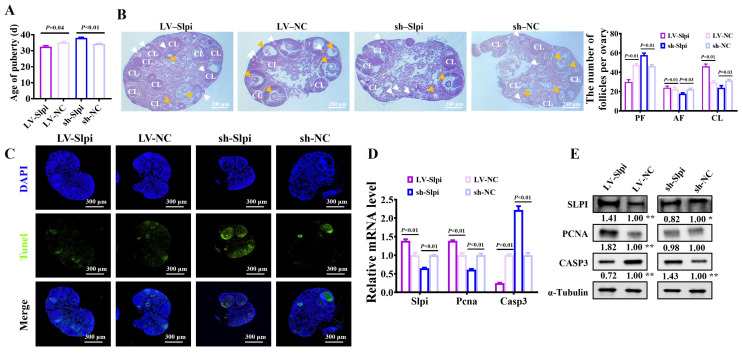
*Slpi* promoted pubertal initiation and inhibited the apoptosis of GCs in mice. (**A**) The ages of pubertal initiation treated with lentiviral vectors overexpressing (LV-*Slpi*) and interfering (sh-*Slpi*) with *Slpi* in mice. (**B**) The number of preantral follicles (PF), antral follicles (AF), and corpus luteum (CL) per ovary in mice treated with LV-*Slpi* and sh-*Slpi*. The white arrow indicates PF, and the yellow arrow indicates AF. Scale bar = 200 μm. (**C**) The apoptosis of follicular GCs treated with LV-*Slpi* and sh-*Slpi*. Scale bar = 300 μm. (**D**) The mRNA levels of *Slpi*, *Pcna*, and *Casp3* in mice treated with LV-*Slpi* and sh-*Slpi*. (**E**) The protein levels of SLPI, PCNA, and CASP3 in mice treated with LV-*Slpi* and sh-*Slpi*. Data are shown as mean ± SEM from three independent biological replicates. *p* values are stated in the figures. *, *p* < 0.05. **, *p* < 0.01.

To further investigate the role of *Slpi* in regulating follicular development in vivo, we intraperitoneally injected *Slpi* overexpression (LV-*Slpi*) and *Slpi* knockdown lentivirus (sh-*Slpi*) into C57BL/6J mic. The mice injected with LV-*Slpi* (32.5 ± 0.7 d) exhibited a significantly earlier onset of puberty compared to the LV-NC control group (35.0 ± 0.8 d, Figure 5A). Compared to the sh-NC controls (34.3 ± 0.5d), mice injected with sh-*Slpi* (38.0 ± 0.5 d) experienced a significant delay in vaginal opening and pubertal initiation. The mice injected with LV-*Slpi* showed a significant decrease in the number of PF and a marked increase in the number of CL. The mice injected with sh-*Slpi* showed a significant increase in the number of PF and a marked decrease in the number of AF and CL (Figure 5B). In mouse ovaries, LV-*Slpi* reduced GC apoptosis, whereas sh-*Slpi* promoted it (Figure 5C). Additionally, we detected the efficiency of LV-*Slpi* and sh-*Slpi*. LV-*Slpi* significantly increased the mRNA (Figure 5D) and protein (Figure 5E) levels of *Slpi* and *Pcna* while lowering those of *Casp3*. sh-*Slpi* showed the opposite results. Together, these data showed that *Slpi* suppressed GC apoptosis and then promoted pubertal initiation in mice.

## 4. Discussion

Puberty represents a critical developmental stage during which female pigs acquire reproductive capacity. The epigenetic regulation mechanism of pubertal initiation mainly focuses on DNA methylation. For instance, Lomniczi et al. [18] found that DNA methylation inhibition disrupts ovulation and delays puberty in rats. However, the mechanism by which histone methylation regulates pubertal initiation remains to be further clarified. The synergistic interaction between GCs and oocytes is essential for pubertal initiation and the maintenance of estrous cycles. As critical components and functional units of ovarian follicles, GCs play a pivotal role in determining follicle fate during puberty [40]. To investigate the role of histone methylation H3K27me3 in porcine follicular development, we treated GCs with an H3K27me3 agonist and inhibitor, respectively. In our study, RNA-seq revealed that H3K27me3 significantly affected the regulation of cell proliferation, apoptotic, and mitotic cell cycle (Figure 1C), as well as estrogen metabolic and female gonad development pathways (Figure 1D). The agonist/inhibitor-treated GCs showed that H3K27me3 significantly suppressed proliferation (Figure 2C) and increased apoptosis (Figure 2E). Furthermore, in vivo experiments using agonist/inhibitor-treated mice showed that H3K27me3 delayed pubertal initiation (Figure 2E). Consistent with previous studies, reduced H3K27me3 levels at the *Ephx2* promoter region led to increased *Ephx2* expression levels, enzymatic activity, and apoptosis in mouse GCs [41].

Ovaries are responsible for hormone secretion, follicular development, and ovulation. Excessive GC apoptosis serves as a primary indicator of follicular atresia and a major contributor to the failure of puberal initiation [42,43]. Epigenetic regulation mainly occurs through DNA methylation, histone post-translational modifications, and RNA editing. In rats, post-translational modification H3K27me3 binds to the *Kiss1* promotor and suppresses its expression before puberty [18]. During puberty, H3K27me3 is erased from the *Kiss1* promoter, while H3K4me3 and H3K27ac are established, thereby enhancing its expression [44]. In rhesus macaques, GATA Zinc Finger Domain Containing 1 inhibits the level of H3K4me3 and suppresses the expression of *Kiss1* during pubertal transition [45]. In human ovaries, elevated H3K27me3 levels at the *CYP11A1* promoter significantly suppress its transcription [46] and accelerate follicular atresia [47]. In our study, we found that the level of H3K27me3 progressively decreased during follicular development (Figure 3A). ChIP-seq analysis was further utilized to obtain genome-wide and time-specific modifications of H3K27me3 at the onset of puberty. Compared to the Pre-puberty stage, In-puberty increased H3K27me3 signaling around the transcriptional start site (Figure 3C). GO analysis of the In- and Pre-puberty groups showed that the downregulated DEGs annotated to gene promoters were significantly enriched in pathways related to sex differentiation, serine-type endopeptidase inhibitor activity, response to external stimulus, and other biological processes (Figure 3D). Compared to the Pre-puberty stage, the *SLPI* promoter region (chr17: 53,166,301–53,167,600) in porcine ovaries showed significantly reduced enrichment of H3K27me3 during the in-pubertal stage (Figure 3E). Therefore, investigating the mechanism by which *SLPI* regulates pubertal initiation, follicular development, and GC function is of great significance.

The *SLPI* promoter region exhibited significantly lower H3K27me3 enrichment in pubertal ovaries compared to pre-pubertal ovaries, suggesting that H3K27me3 regulated pubertal initiation by repressing *SLPI* transcription. *SLPI* is a member of the whey-acidic protein family [48], characterized by a boomerang-like shape and two domains with similar architecture, connected by four disulfide bridges [49]. *SLPI* mediates a variety of biological processes, including cell proliferation and apoptosis, repairing reaction, and immune response. Numerous studies indicate that *TGF-β1* prevents premature luteinization, downregulates the expression of the luteinization-related gene *MMP1*, and promotes human type I collagen deposition [50,51]. The *SLPI* can potentially affect various signaling pathways, including NF-κB, by preventing *p65* interaction with the *NF-κB* consensus region at physiological nuclear concentrations [52]. In our study, the transcription level of the *SLPI* gene was significantly lower in immature follicles than in mature follicles (Figure 4A). Downregulating H3K27me3 levels in porcine ovarian GCs significantly increased *SLPI* transcription, while upregulation reduced it (Figure 4B). *SLPI* significantly promoted GC proliferation (Figure 4F) and inhibited apoptosis (Figure 4H). In mice, *Slpi* overexpression accelerated pubertal initiation (Figure 5A) and follicular development (Figure 5B), while inhibiting the GC apoptosis in the ovaries (Figure 5C). Other researchers also confirm that *SLPI* suppresses *MMP9* expression [53], and both *MMP2* and *MMP9* inhibit GC proliferation and promote apoptosis [54]. Moreover, the elevation of H3K27me3 by GSK-J4 reduces tumor size and weight in the OVA250X mouse model of ovarian cancer [55]. H3K27me3 accumulation aggravates GC senescence in H_2_O_2_-induced ovarian aging [56]. H3K27me3 suppresses porcine GC proliferation and promotes ferroptosis via the *FoxO1*-*CYP1A1* axis [20]. Given these findings, the H3K27me3–*SLPI* axis identified in our study may represent a novel target for pubertal assessment in gilts, a tool for improving reproductive efficiency in livestock production, and a therapeutic target for reproductive disorders.

Using mice instead of pigs for in vivo experiments is a limitation of this study. Pubertal markers (e.g., vulvar appearance, vaginal opening, and follicle development) are similar between pigs and mice. H3K27me3 regulation is also highly conserved across mammals, including pigs [20], mice [19], rats [18], and humans [21]. The age of pubertal initiation in the Landrace × Large White sows used in this study is approximately 210 days, making lentiviral experiments in pigs technically challenging and expensive. Mice have been widely used as a research model for pubertal initiation in mammals. For example, lncRNA-IFFD acts as a miR-370 sponge to increase *GLI1* expression, promoting apoptosis and suppressing proliferation and E2 secretion in porcine GCs, thereby blocking pubertal onset in mice [32]. In Davisdale ewes, the leptin receptor mutation delays puberty and causes infertility. Nonetheless, we suggest future experiments in pigs using H3K27me3 agonists/inhibitors and lentiviral *SLPI* treatment to further validate the H3K27me3-*SLPI* axis and its impact on pubertal timing. Furthermore, our results suggested that H3K27me3 might regulate *SLPI* transcription by influencing the binding of the transcription factor SOX4. *SOX4*, a stress-responsive epigenetic regulator in GCs [57], promotes GC proliferation and inhibits apoptosis in humans [58]. The precise molecular mechanism remains unclear. Therefore, cellular experiments are needed to clarify how H3K27me3 and SOX4 coordinate to regulate *SLPI* expression. Specifically, we propose chromatin accessibility assays to assess the impact of H3K27me3 on the *SLPI* promoter, as well as ChIP-PCR and H3K27me3 agonist/inhibitor treatments to examine SOX4 binding to the *SLPI* promoter. Rescue experiments in GCs are also needed (e.g., H3K27me3 elevation with *SLPI* overexpression, or H3K27me3 reduction with *SLPI* knockdown), which could further elucidate how the H3K27me3-SOX4-*SLPI* axis regulates porcine follicular development and pubertal initiation. Moreover, the relatively small sample size in this study may limit statistical power, and our findings should be validated in larger cohorts in the future.

## 5. Conclusions

**Figure 6:**
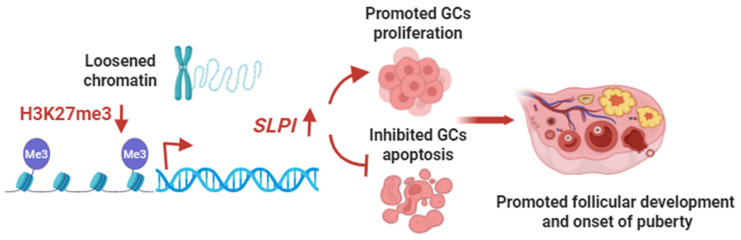
Schematic model of the H3K27me3-*SLPI* axis. The loss of H3K27me3 at the promoter of *SLPI* enhanced *SLPI* transcription, which in turn promoted cell proliferation, suppressed apoptosis, and accelerated follicular development and pubertal initiation.

In conclusion, our findings demonstrated that the diminished enrichment of H3K27me3 at the *SLPI* promoter enhanced its transcriptional activity by increasing chromatin accessibility, which in turn promoted porcine GC proliferation and suppressed apoptosis in vitro. Furthermore, *Slpi* accelerated pubertal initiation in mice (Figure 6).

## Figures and Tables

**Table 1:** Primers used for qRT-PCR.

Primer Name	Sequence (5′–3′)
GAPDH-pig-F	GGACTCATGACCACGGTCCAT
GAPDH-pig-R	TCAGATCCACAACCGACACGT
SLPI-pig-F	TCCGCAAGTATCTCGGCAAC
SLPI-pig-R	TTGGACTTTGTGGGCAGTCG
PCNA-pig-F	ATGCAGACACCTTGGCACTA
PCNA-pig-R	ACAGCTGTACTCTTGTTCTGGA
STAR-pig-F	CGACGTTTAAGCTGTGTGCT
STAR-pig-R	ATCCATGACCCTGAGGTTGGA
BCL2-pig-F	GATGCCTTTGTGGAGCTGTATG
BCL2-pig-R	CCCGTGGACTTCACTTATGG
CASP3-pig-F	ACATGGAAGCAAATCAATGGAC
CASP3-pig-R	TGCAGCATCCACATCTGTACC
CASP7-pig-F	CGTTGCGGCTTTACTTTCGCT
CASP7-pig-R	ACTCCAGGACAGCTCCTTGAAGA
CASP9-pig-F	GCTGAACCGTGAGCTTTTCA
CASP9-pig-R	CCTGGCCTGTGTCCTCTAAG
BID-pig-F	ACGAGCGCATCACAAACCTA
BID-pig-R	GCCTCCTGGCTCTCAGAATC
GAPDH-mouse-F	GGTCCCAGCTTAGGTTCATCA
GAPDH-mouse-R	CCAATACGGCCAAATCCGTT
SLPI-mouse-F	AAGCAGAGGTGCTGCCAAGATG
SLPI-mouse-R	TCTGGCAGACATTGGGAGGGTT
PCNA-mouse-F	CAAGTGGAGAGCTTGGCAATGG
PCNA-mouse-R	GCAAACGTTAGGTGAACAGGCTC
CASP3-mouse-F	GGAGTCTGACTGGAAAGCCGAA
CASP3-mouse-R	CTTCTGGCAAGCCATCTCCTCA

## Data Availability

The RNA-seq (accession number: PRJNA1373485) and ChIP-seq (accession number: PRJNA1372938) data of this study have been deposited in the NCBI BioProject database. The original contributions presented in this study are included in the article. Further inquiries can be directed to the corresponding authors.

## Abbreviations

H3K27me3, Histone H3 lysine 27 trimethylation. GC, granulosa cell. *SLPI*, secretory leukocyte peptidase inhibitor. ChIP-seq, chromatin Immunoprecipitation sequencing. MMP, matrix metalloproteinase. PBS, phosphate-buffered saline. DEGs, differentially expressed genes. qRT-PCR, quantitative real-time PCR. FITC, fluorescein isothiocyanate. PI, propidium iodide. HE, hematoxylin and eosin. PF, preantral follicles. AF, antral follicles. CL, corpora lutea. E2, estrogen. OE-*SLPI*, overexpression vectors of *SLPI*. si-*SLPI*, small interfering RNA of *SLPI*. LV-*Slpi*, overexpression lentivirus of *Slpi*. sh-*Slpi*, knockdown lentivirus of *Slpi*.
